# Efficacy of Jia Wei Shoutai Wan Combined With Dydrogesterone in the Treatment of Threatened Abortion Complicated With Endometrial Cavity Fluid: A Prospective, Single‐Center, Randomized Controlled Trial

**DOI:** 10.1155/ije/5226490

**Published:** 2026-04-05

**Authors:** Yongrong Zhang, Huan Wei, Hui Li, Yu Zhang, Fang Jia

**Affiliations:** ^1^ Obstetrics and Gynecology Department, Mianyang Maternal and Child Health-Care Hospital, Mianyang, 621000, Sichuan, China, scu.edu.cn; ^2^ Department of Traditional Chinese Medicine, Mianyang Maternal and Child Health-Care Hospital, Mianyang, 621000, Sichuan, China, scu.edu.cn

**Keywords:** abortion, Chinese traditional, dydrogesterone, medicine, threatened, treatment outcome, uterine diseases

## Abstract

**Objective:**

This study aimed to evaluate the clinical efficacy of Jia Wei Shoutai Wan (JWSTW) combined with dydrogesterone in patients with threatened abortion (TA) complicated by endometrial cavity fluid (ECF).

**Methods:**

This was a prospective, single‐center, randomized controlled trial. A total of 130 patients with TA and ECF admitted to our hospital from January to November 2022 were screened. Thirteen patients did not meet the inclusion criteria, and five refused to participate, leaving 112 eligible participants. Using a random number table, patients were assigned to a control group (dydrogesterone alone, *n* = 56) or a combination group (dydrogesterone plus JWSTW, *n* = 56). Both groups received continuous treatment for 14 days. During follow‐up, 3 patients withdrew and 6 were excluded, resulting in 103 cases included in the final analysis (control group, *n* = 50; combination group, *n* = 53). The primary outcomes were ECF area and Traditional Chinese Medicine (TCM) syndrome scores (vaginal bleeding, lower abdominal pain, fatigue and tiredness, knee soreness, and lumbago) after 14 days of treatment. Secondary outcomes included serum levels of progesterone (P), β‐human chorionic gonadotropin (β‐HCG), and estradiol (E2); coagulation indices [D‐dimer (D‐D), fibrinogen (FIB), and prothrombin time (PT)]; clinical efficacy; and adverse reactions.

**Results:**

After treatment, the combination group showed significantly lower TCM symptom scores and a smaller ECF area (*p* < 0.05). Serum levels of P, β‐HCG, and E2 were higher in the combination group, while D‐D and FIB levels were lower and PT was longer compared with the control group (*p* < 0.05). Adverse reactions were monitored, including premature rupture of membranes, postpartum hemorrhage, placental adhesion, preterm delivery, and placenta previa. The total incidence was 8.00% in the control group (4/50) and 3.77% in the combination group (2/53), with no statistically significant difference between the groups (*p* = 0.360). The overall clinical efficacy of the combination group was superior to that of the control group (*p* < 0.05).

**Conclusion:**

JWSTW combined with dydrogesterone may be beneficial for treating TA with ECF by improving clinical symptoms, optimizing hormone and coagulation profiles, and reducing ECF without increasing adverse reactions.

## 1. Introduction

Threatened abortion (TA) is a common complication occurring during early pregnancy, particularly between 8 and 12 gestational weeks while estrogen and progesterone (P) secretion shifts from the corpus luteum to the placental [1]. If not treated properly, TA may result in miscarriage [2]. TA’s incidence has been increasing in recent years, imposing substantial psychological and emotional stress on pregnant women and their family members [3]. Endometrial cavity fluid (ECF) refers to the abnormal accumulation of fluid within the endometrial cavity [4]. It is closely related to adverse pregnancy outcomes such as implantation failure, early miscarriage, and intrauterine infection [5]. ECF may impair endometrial receptivity and trophoblast invasion, contributing to poor embryo implantation and pregnancy maintenance [6, 7]. At present, the management of TA complicated with ECF mainly relies on P supplementation, yet its effectiveness varies among individuals. Therefore, identifying adjunctive strategies to improve pregnancy outcomes and reduce ECF recurrence remains a significant clinical need.

Various Chinese herbal medicines have demonstrated favorable protective effects against TA [8]. Shoutai Wan (STW), a traditional Chinese medicine (TCM) formula, has long been utilized to treat TA due to its effects of calming the fetus and tonifying the kidney [9]. STW may exert its therapeutic actions through multiple mechanisms involving hormonal regulation, anti‐inflammation, and endometrial protection [10]. A previous study has reported that Shoutai pill (STP, also known as STW) effectively enhances endometrial receptivity [11] and improves embryo implantation rate [12]. It has also been applied to enhance endometrial acceptability and promote pregnancy outcomes [13]. The modified formula Jia Wei Shoutai Wan (JWSTW) is an evolved version of STW that reinforces its therapeutic efficacy and has shown clinical benefits in reducing the embryo loss rate and improving litter size in animal models [14, 15]. In clinical applications, STW or JWSTW has achieved good results in treating TA and other pregnancy‐related complications.

Derived from the classical Chinese medical text *Yixue Zhongzhong Canxi Lu*, STW is traditionally prescribed for fetal restlessness and sliding fetus, with the treatment principles of tonifying the kidney, strengthening qi, and stabilizing the Chong and Ren meridians. These classical theories correspond with modern medical concepts such as supporting luteal function, regulating endocrine secretion, improving uterine blood flow, and enhancing endometrial receptivity. The major herbal components—*Cuscuta chinensis* (Tusizi), *Taxillus chinensis* (Sangjisheng), *Eucommia ulmoides* (Duzhong), and *Dipsacus asper* (Xuduan)—have been shown to regulate sex hormones, enhance corpus luteum function, alleviate hypercoagulability, improve microcirculation, and modulate immune balance [16]. These multitarget effects reflect the integrative advantages of JWSTW in managing early pregnancy complications.

Modern pharmacological evidence further supports these mechanisms. Liang et al. found that STW improved embryo survival in a mouse model of recurrent spontaneous abortion by regulating aerobic glycolysis of trophoblast cells via the HK2/PKM2/LDHA pathway [17]. Yang et al. used a network pharmacology strategy to reveal that STW acts through multiple targets related to inflammation, oxidative stress, and hormone signaling [18]. Clinically, Liu et al. confirmed that STW combined with luteal support significantly reduced miscarriage rates in women undergoing IVF–FET cycles [19]. These findings provide a robust scientific rationale for the clinical application of JWSTW in early pregnancy complications.

Dydrogesterone, an orally active synthetic progestogen [20], remains one of the most widely used hormonal agents in early pregnancy. It effectively prevents miscarriage in women with TA by supporting luteal function and stabilizing endometrial development, thereby lowering the overall incidence of pregnancy loss [21, 22]. However, dydrogesterone mainly targets hormonal pathways and has limited effects on coagulation, immune modulation, and endometrial microcirculation. In addition, mild side effects—such as nausea, dizziness, or breast tenderness—have occasionally been observed in clinical practice [23]. In contrast, JWSTW offers distinct multitarget advantages. Through its combination of kidney‐tonifying and qi‐strengthening herbs, JWSTW not only supports progesterone synthesis and luteal activity but also enhances endometrial receptivity, modulates immune and inflammatory responses, stabilizes coagulation, and improves uterine microcirculation. Mechanistically, it exerts antioxidative and anti‐inflammatory effects that promote embryo implantation and maintain a stable maternal–fetal interface [16–18]. These synergistic actions are believed to complement the hormonal effects of dydrogesterone and potentially overcome its limitations in endometrial and vascular regulation. The combination of STP and dydrogesterone has been reported to achieve superior therapeutic outcomes and favorable safety profiles in the management TA [24].

Taken together, JWSTW demonstrates promising therapeutic potential by integrating TCM principles with modern pharmacological actions. However, limited evidence is available regarding its efficacy in TA complicated by ECF. Therefore, this study aimed to evaluate the clinical effects of JWSTW combined with dydrogesterone on hormonal and coagulation profiles, ECF, and overall therapeutic outcomes in patients with TA complicated by ECF.

## 2. Materials and Methods

### 2.1. Ethics Statement

The study was reviewed and approved by the Ethics Committee of Mianyang Maternal and Child Health‐Care Hospital and conducted in accordance with the Declaration of Helsinki. All participants were fully informed about the study and provided written informed consent prior to enrollment.

### 2.2. General Data

**FIGURE 1:**
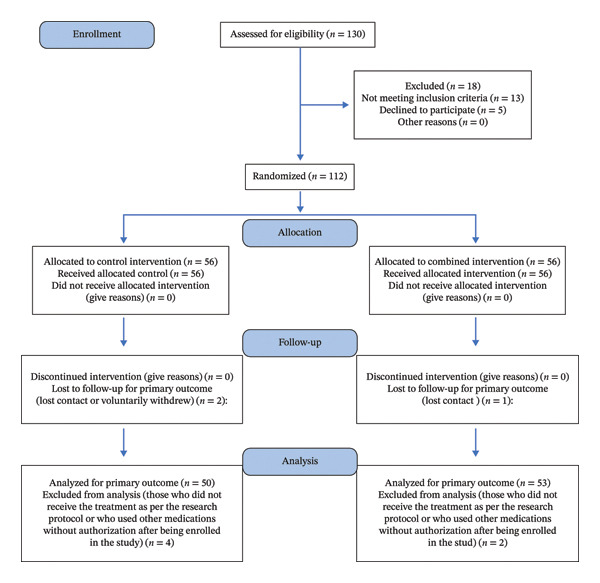
Consort flowchart illustrating the patient recruitment, allocation, follow‐up, and analysis.

This study was designed as a prospective, single‐center, randomized controlled trial. Using a consecutive sampling method, a total of 130 patients diagnosed with TA complicated by ECF who were admitted to Mianyang Maternal and Child Health‐Care Hospital between January and November 2022 were initially screened. Among them, 13 patients did not meet the inclusion criteria, and five declined to participate, leaving 112 eligible participants for enrollment. All eligible patients were randomly assigned using a random number table method into two groups: the control group (treated with dydrogesterone, *n* = 56) and the combination group (treated with JWSTW in addition to dydrogesterone, *n* = 56). During the study period, three patients were lost to follow‐up and six were excluded due to protocol deviations, resulting in 103 patients who completed the study and were included in the final analysis (50 in the control group and 53 in the combination group) (Figure 1).

### 2.3. Diagnostic Criteria for Early TA

The diagnosis was established according to the criteria described in *Obstetrics and Gynecology*. Patients presented with amenorrhea, milder vaginal bleeding, lower abdomen discomfort, pelvic heaviness, or lumbosacral soreness. The uterine size was consistent with the gestational age, and the cervical os remained closed. Pregnancy tests were positive, and ultrasonography revealed a gestational sac with visible fetal cardiac activity and fetal movement.

### 2.4. Diagnostic Criteria for ECF

Ultrasonography displayed crescent‐shaped, triangular, or circular anechoic areas of fluid accumulation within the uterine cavity, between the uterine wall and the gestational sac, or between the uterine wall and the placenta.

### 2.5. Syndrome Differentiation Criteria

Based on the *Gynecology of Traditional Chinese Medicine*, the diagnostic criteria for “Spleen‐Kidney Qi Deficiency Syndrome” were applied. Clinical manifestations included lumbosacral soreness and abdominal pain with a sensation of downward pressure during pregnancy, accompanied by scanty vaginal bleeding of light or dark color and thin consistency. Additional symptoms included fatigue, lassitude of the limbs, dizziness, tinnitus, frequent urination, and nocturia. The tongue was pale with a white coating, and the pulse was deep, slippery, and weak in the chi region.

### 2.6. Inclusion Criteria

Patients who met the above diagnostic and syndrome differentiation criteria; aged 20–45 years, gestational age < 12 weeks; confirmed intrauterine singleton viable pregnancy; ultrasound evidence of ECF.

### 2.7. Exclusion Criteria

Patients were excluded if they had confirmed ectopic pregnancy; concomitant ovarian tumors or uterine malformations; vaginal bleeding caused by other reasons; a history of recurrent miscarriage; gestational hypertension or diabetes mellitus; prior use of other progestational or miscarriage‐preventive drugs; known allergy to dydrogesterone or any component of JWSTW; severe heart, liver, kidney, or hematopoietic diseases; or contraindications to either dydrogesterone or herbal miscarriage‐prevention therapy.

### 2.8. Elimination Criteria

Patients who, after enrollment, failed to adhere to the study protocol or used unauthorized medications were excluded from the analysis. Those who developed severe adverse events or complications making continuation of the original treatment regimen inappropriate were also eliminated.

### 2.9. Dropout Criteria

Patients who were lost to follow‐up, unable to complete the study, or voluntarily withdrew from the trial at the request of themselves or their families were classified as dropouts.

### 2.10. Sample Size Estimation

The sample size for this study was estimated using the statistical efficiency method in G^∗^Power 3.1.9.7 (University of Düsseldorf, Germany). The primary outcome variables were continuous measures (e.g., TCM syndrome scores and ECF area) tested for superiority. Because no pre‐experimental data exactly matching the present intervention (JWSTW plus dydrogesterone) and population (TA with ECF) were available to precisely calculate the effect size, the estimation was mainly based on clinical relevance and data from comparable studies.

The effect size (Cohen’s d) was set at 0.8, representing a large effect. This assumption was grounded on the expectation that the combination therapy would yield clinically meaningful improvement in key efficacy endpoints compared with dydrogesterone alone. Previous studies of similar TCM interventions have reported medium‐to‐large effect sizes (*d* = 0.5–0.8), supporting this parameter choice [25, 26].

Statistical parameters were defined as two‐tailed *α* = 0.05 and 1−*β* = 0.95. The allocation ratio between the control (*N*_1_) and combination (*N*_2_) groups was 1:1. The calculated sample size was *N*_1_ = 42 and *N*_2_ = 42, yielding a total of 84 participants. Allowing for an anticipated dropout rate of approximately 20%, the initial enrollment target was 130 patients. Among them, 13 did not meet the inclusion criteria, and five declined participation. During the study, three participants were lost to follow‐up and six were excluded, resulting in 103 patients (50 in the control group and 53 in the combination group) included in the final analysis. The achieved sample size met the requirements for statistical validity of all planned analyses.

### 2.11. Randomization and Allocation Concealment

Random sequence generation: An independent statistician who was not involved in patient recruitment, group assignment, or treatment procedures generated a random allocation sequence using the random number generation function of GraphPad Prism 8.0 software. The sequence was produced at a 1:1 ratio, with sequence A representing the control group and sequence B representing the combination group. Allocation concealment: The randomization sequence was placed into sequentially numbered, opaque, sealed envelopes. Each envelope was labeled externally with only its corresponding serial number (from 1 to 112). All envelopes were securely stored and managed by an independent pharmacist who was not involved in the study’s clinical operations. Implementation of group assignment: When an eligible participant signed the informed consent form and was officially enrolled, the recruiting physician opened the next envelope in numerical order and assigned the patient to the corresponding group based on the allocation card inside. Blinding: This study employed an assessor‐blinded design. Due to the inherent differences in the intervention regimens between the two groups (Western medicine versus Western medicine combined with a traditional Chinese herbal formula and external application), blinding of patients and treating nurses was not feasible. To minimize bias, the following measures were implemented: Blinding of outcome assessors: Laboratory technicians responsible for measuring serum indicators (e.g., P, β‐human chorionic gonadotropin [β‐HCG], estradiol [E2], D‐dimer [D‐D], fibrinogen [FIB], and prothrombin time [PT]), ultrasound physicians measuring ECF area, and research staff recording TCM syndrome scores were all blinded to group allocation. Blinding of data analysts: During the statistical analysis phase, data analysts were unaware of the specific treatment assignments corresponding to groups A and B until all analyses were completed. Patient communication strategy: During the informed consent process, participants were informed that the study aimed to compare the efficacy of two pregnancy‐preserving regimens without emphasizing the differences between them, thereby reducing expectancy bias.

### 2.12. Methods

#### 2.12.1. Control Group

Patients in the control group received dydrogesterone therapy. Dydrogesterone tablets (Abbott Biologicals B.V., State Drug Administration: H20130110, specification: 10 mg/tablet) were administered orally. On the first day, patients received 40 mg once daily; from the second day onward, 10 mg per dose, twice daily.

#### 2.12.2. Combination Group

On the basis of dydrogesterone therapy, patients in the combination group additionally received JWSTW. Herbal formula composition: *C. chinensis* (Lam.) Sieb. ex Spreng. (*Tusizi*, 15 g), *T. chinensis* (DC.) Danser (*Sangjisheng*, 15 g), *E. ulmoides* Oliv. (*Duzhong*, 15 g), *D. asper* Wall. ex Henry (*Xuduan*, 15 g), and *Glycyrrhiza uralensis* Fisch. (*Zhigancao*, 6 g). For syndrome differentiation and treatment modification: Kidney deficiency with blood heat: add *Rehmannia glutinosa* Libosch. (*Sheng Dihuang*, raw rehmannia), *Rehmannia glutinosa* Praep. (*Shu Dihuang*, prepared rehmannia), *Paeonia lactiflora* Pall. (*Baishao*), *Ligustrum lucidum* Ait. (*Nüzhenzi*), and *Eclipta prostrata* L. (*Mohanlian*). Spleen–kidney deficiency: Kidney deficiency with blood stasis: add *Astragalus membranaceus* (*Huangqi*), *Angelica sinensis* (Oliv.) Diels (*Danggui*), *Codonopsis pilosula* (Franch.) Nannf. (*Dangshen*) and *Rehmannia glutinosa Praep.* (*Shu Dihuang*). The decoction was prepared daily using an automatic decocting machine in the TCM Pharmacy of Mianyang Maternal and Child Health‐Care Hospital or provided as concentrated granules. Patients were instructed to take the decoction warm, three times daily. Acupoint application therapy: Application sites: Shenshu (BL23) and Zusanli (ST36). Topical herbal paste ingredients: *C. chinensis* (Lam.) Sieb. ex Spreng. 3 g, *T. chinensis* (DC.) Danser 2 g, *D. asper* Wall. ex Henry 2 g, *Astragalus membranaceus* (Fisch.) Bge. 3 g, and *Psoralea corylifolia* L. (*Buguzhi*) 2 g. The medicated patches were applied once every other day.

Both groups received continuous treatment for 14 days.

### 2.13. Efficacy Observation

In accordance with the CONSORT guidelines, the following primary and secondary outcome measures were established for this study: Primary outcomes: (1) The area of ECF after 14 days of treatment and (2) The total TCM syndrome score after 14 days of treatment, which included the following symptom domains: vaginal bleeding, lower abdominal distension and pain, fatigue and weakness, soreness of the knees, and lumbago. Secondary outcomes: (1) Serum endocrine hormone levels: P, β‐HCG, and E2; (2) Coagulation function indices: D‐D, FIB, and PT; (3) Overall clinical efficacy rate, assessed according to the *Standards for the Diagnosis and Efficacy Evaluation of TCM Diseases and Syndromes*; and (4) Incidence of adverse reactions. Details of these outcome assessments are as follows:

1. TCM syndrome score: Prior to and after treatment, TCM symptom scores were evaluated in both groups, covering the following five symptom domains: vaginal bleeding, lower abdominal distension and pain, fatigue and weakness, soreness of the knees, and lumbago. Each item was scored on a 0–3 scale, with higher scores indicating greater symptom severity.
2. Hormone and coagulation function testing: Prior to and after treatment, 4 mL of fasting venous blood was collected for each patient in the morning. Serum levels of P, β‐HCG, and E2 were measured by the chemiluminescence immunoassay method. D‐D, FIB, and PT were measured by the ACL TOP automated coagulation analyzer (Beckman Coulter, USA).
3. Measurement of ECF area: The ECF area was measured using B‐mode ultrasonography (Philips Affiniti 50 color Doppler diagnostic system). The anteroposterior uterine diameter, gestational sac dimensions, and the ECF size were measured. The longest diameter and the perpendicular diameter of the effusion were recorded as length and width, respectively, and the ECF area was calculated as length × width.
4. Evaluation of clinical efficacy: Clinical efficacy was assessed according to the Standards for the Diagnosis and Efficacy Evaluation of TCM Diseases and Syndromes and the Guidelines for Clinical Research of New Chinese Medicines. The criteria were as follows: (1) Cured: All clinical symptoms resolved; ultrasound showed fetal development consistent with gestational age and no detectable ECF. (2) Significantly effective: Clinical symptoms significantly alleviated; ultrasound showed fetal development consistent with gestational age and *a* > 60% reduction in ECF area. (3) Effective: Clinical symptoms moderately improved; ultrasound showed fetal development consistent with gestational age and a 30%–60% reduction in ECF area. (4) Ineffective: Did not meet the above criteria. The total effective rate was calculated as (cured cases + significantly effective cases + effective cases)/total cases × 100%.
5. Adverse events: All adverse events occurring during the treatment period were documented and analyzed in both groups.

### 2.14. Safety and Risk Management

In this study, all treatment‐related adverse reactions and side effects were defined as key safety evaluation indicators and were closely monitored throughout the trial. The specific measures were as follows:

#### 2.14.1. Active Monitoring and Documentation

During each follow‐up visit, investigators actively inquired about and recorded any potential adverse reactions through patient interviews, physical examinations, and laboratory tests. Adverse events included, but were not limited to, nausea, vomiting, dizziness, rash, and abnormalities in liver function. Each event was evaluated for its potential causal relationship with the study medication.

#### 2.14.2. Risk Prevention

To minimize possible gastrointestinal discomfort caused by the herbal decoction, patients were instructed to take the medication warm and after meals.

#### 2.14.3. Criteria for Treatment Discontinuation

Study medication was to be immediately discontinued, and appropriate clinical management and follow‐up were provided if any of the following occurred: occurrence of a serious adverse event; worsening signs of TA, such as intensified uterine contractions or significantly increased vaginal bleeding, requiring treatment modification; or voluntary withdrawal of the patient from the study.

### 2.15. Statistical Analysis

Data were processed in GraphPad Prism 8.0 (GraphPad Software, La Jolla, CA, USA). Distributional assumptions were assessed with the Kolmogorov–Smirnov test. Variables conforming to normality are reported as mean ± standard deviation (SD); between‐group comparisons used the independent‐samples *t* test, and within‐group pre‐/post‐treatment changes used the paired *t* test. Non‐normally distributed data are summarized as median (*Q*_1_, *Q*_3_); between‐group differences were examined with the Mann–Whitney test, and within‐group changes with the Wilcoxon matched‐pairs signed‐rank test. Categorical variables were analyzed using the chi‐square test and expressed as percentages (%). Statistical significance was set at *p* < 0.05.

## 3. Results

### 3.1. Baseline Characteristics

A total of 103 patients were included in the final analysis (control group, *n* = 50; combination group, *n* = 53). As shown in Supporting Table 1, there were no significant differences between the two groups in any of the collected baseline variables, including age, gestational week, obstetric history (gravidity, parity, and history of miscarriage), or duration of vaginal bleeding (*p* > 0.05). These findings indicate that the randomization process achieved good balance between the groups, ensuring comparability. Furthermore, the study’s rigorous randomization and allocation concealment procedures, along with blinded outcome assessment, effectively minimized potential confounding bias throughout the research process.

### 3.2. TCM Symptom Scores

No significant differences were observed in the TCM symptom scores for vaginal bleeding, lower abdominal pain, fatigue and lassitude, knee soreness, and lumbago between the two groups before treatment (*p* > 0.05). After treatment, all symptom scores decreased in both groups, with significantly lower scores observed in the combination group compared with the control group (*p* < 0.05) (Table 1).

**TABLE 1:** Comparison of TCM symptom scores prior to and after treatment between the two groups.

	Control group (*n* = 50)	Combination group (*n* = 53)	*p*
Vaginal bleeding			
Before treatment	2.12 ± 0.52	2.26 ± 0.55	0.189
After treatment	1.12 ± 0.32^∗^	1.02 ± 0.14^∗^	0.040
Lower abdominal pain			
Before treatment	2.50 ± 0.57	2.53 ± 0.54	0.784
After treatment	1.14 ± 0.35^∗^	0.94 ± 0.23^∗^	< 0.001
Fatigue and lassitude			
Before treatment	2.24 ± 0.47	2.43 ± 0.50	0.098
After treatment	1.16 ± 0.37^∗^	0.91 ± 0.29^∗^	< 0.001
Knee soreness			
Before treatment	2.38 ± 0.52	2.40 ± 0.49	0.841
After treatment	1.24 ± 0.43^∗^	0.87 ± 0.34^∗^	< 0.001
Lumbago			
Before treatment	2.32 ± 0.47	2.34 ± 0.47	0.830
After treatment	1.14 ± 0.35^∗^	0.85 ± 0.36^∗^	< 0.001

^∗^ *p* < 0.05 versus the same group before treatment.

### 3.3. Endocrine Hormone Levels

No significant differences were found in serum levels of P, β‐HCG, and E2 between the two groups prior to treatment (*p* > 0.05). Following treatment, these hormone levels increased in both groups, and the combination group showed significantly higher levels than the control group (*p* < 0.05) (Table 2).

**TABLE 2:** Comparison of endocrine hormone levels prior to and after treatment between the two groups.

	Control group (*n* = 50)	Combination group (*n* = 53)	*p*
P (ng/mL)			
Before treatment	19.62 ± 2.14	18.96 ± 2.32	0.137
After treatment	32.55 ± 4.23^∗^	39.41 ± 4.58^∗^	< 0.001
β‐HCG (mIU/mL)			
Before treatment	12,268.26 ± 2337.39	12,315.41 ± 2227.03	0.917
After treatment	63,152.47 ± 3817.55^∗^	78,161.20 ± 4085.93^∗^	< 0.001
E2 (pg/mL)			
Before treatment	271.87 ± 30.75	274.66 ± 30.18	0.643
After treatment	398.15 ± 35.25^∗^	468.29 ± 39.80^∗^	< 0.001

^∗^ *p* < 0.05 versus the same group before treatment.

### 3.4. Adverse Reactions

Adverse reactions were monitored, including premature rupture of membranes, postpartum hemorrhage, placental adhesion, preterm delivery, and placenta previa. The total incidence of adverse reactions was 8.00% in the control group and 3.77% in the combination group. There was no significant difference in the incidence of adverse events between the two groups (*p* < 0.05) (Table 3).

**TABLE 3:** Comparison of adverse reactions between the two groups.

	Control group (*n* = 50)	Combination group (*n* = 53)	*p*
Premature rupture of membranes	2 (4.00)	0 (0.00)	—
Postpartum hemorrhage	0 (0.00)	1 (1.89)	—
Placental adhesion	0 (0.00)	1 (1.89)	—
Premature delivery	1 (2.00)	0 (0.00)	—
Placenta previa	1 (2.00)	0 (0.00)	—
Total incidence rate	4 (8.00)	2 (3.77)	0.360

### 3.5. ECF Area

Prior to treatment, the mean ECF area was 363.02 ± 129.89 mm^2^ in the control group and 338.57 ± 127.22 mm^2^ in the combination group, with no significant difference between them (*p* > 0.05). Following treatment, the ECF area markedly decreased in both groups to 182.96 ± 87.63 and 106.67 ± 72.70 mm^2^, respectively, and the reduction was significantly greater in the combination group (*p* < 0.05) (Table 4).

**TABLE 4:** Comparison of ECF area prior to and after treatment between the two groups.

ECF area (mm^2^)	Control group (*n* = 50)	Combination group (*n* = 53)	*p*
Before treatment	363.02 ± 129.89	338.57 ± 127.22	0.337
After treatment	182.96 ± 87.63^∗^	106.67 ± 72.70^∗^	< 0.001

^∗^ *p* < 0.05 versus the same group before treatment.

### 3.6. Coagulation Indicators

Prior to treatment, no significant differences were found in D‐D, FIB, and PT levels between the two groups (*p* > 0.05). After treatment, D‐D and FIB were reduced while PT was elevated in both groups, and compared with the control group, the combination group exhibited lower D‐D and FIB levels and higher PT values (*p* < 0.05) (Table 5).

**TABLE 5:** Comparison of coagulation factor indicators prior to and after treatment between the two groups.

	Control group (*n* = 50)	Combination group (*n* = 53)	*p*
D‐D (μg·L^−1^)			
Before treatment	152.39 ± 19.95	151.24 ± 19.48	0.768
After treatment	133.84 ± 15.58^∗^	118.75 ± 12.07^∗^	< 0.001
FIB (g·L^−1^)			
Before treatment	3.88 ± 0.73	3.80 ± 0.78	0.593
After treatment	3.08 ± 0.63^∗^	2.32 ± 0.62^∗^	< 0.001
PT (s)			
Before treatment	11.32 ± 0.86	11.29 ± 0.97	0.869
After treatment	12.34 ± 0.68^∗^	13.22 ± 0.62^∗^	< 0.001

^∗^ *p* < 0.05 versus the same group before treatment.

### 3.7. Efficacy

The total effective rate was 84.00% in the control group and 98.11% in the combination group. The treatment efficacy in the combination group was significantly higher than that in the control group (*p* < 0.05) (Table 6).

**TABLE 6:** Comparison of efficacy after treatment between the two groups.

	Control group (*n* = 50)	Combination group (*n* = 53)	*p*
Cured	2 (4.00)	7 (13.21)	—
Significantly effective	13 (26.00)	33 (62.26)	—
Effective	27 (54.00)	12 (22.64)	—
Ineffective	8 (16.00)	1 (1.89)	—
Total effective rate	42 (84.00)	52 (98.11)	0.012

## 4. Discussion

TA is characterized by vaginal bleeding and uterine cramping during early pregnancy [27] and remains a major cause of early pregnancy loss. This study evaluated the clinical efficacy of JWSTW combined with dydrogesterone in patients with TA complicated by ECF. To our knowledge, this is one of the few clinical studies specifically addressing this combined therapeutic approach in this patient population.

Our findings demonstrate that JWSTW combined with dydrogesterone significantly alleviated TCM‐related clinical symptoms such as vaginal bleeding, abdominal pain, fatigue, knee soreness, and lumbago. The improvement was more pronounced in the combination group than in the dydrogesterone monotherapy group. These results are consistent with previous evidence showing that traditional STW improves uterine function and pregnancy maintenance through endocrine modulation and stabilization of the endometrial environment [16]. Additionally, the combined regimen effectively enhanced serum levels of P, β‐HCG, and E2 in line with previous studies reporting that STW can upregulate P and estrogen receptor expression [12].

The incidence of adverse reactions was low in both groups, with no statistically significant difference, suggesting that the combination of JWSTW and dydrogesterone is generally safe and well tolerated. This aligns with previous reports that STW is associated with minimal side effects when used during early pregnancy [19]. Furthermore, dydrogesterone monotherapy has also been shown to reduce miscarriage rates with minimal adverse effects [23]. The concurrent use of STW and dydrogesterone may thus exert synergistic effects in reducing embryo loss and improving pregnancy outcomes [17].

Another important finding is that the combination therapy promoted the absorption of ECF, as reflected by a significant reduction in ECF area. Since ECF is often associated with inflammation, coagulation imbalance, and impaired implantation, its reduction may indicate improved microcirculatory function and endometrial receptivity. In line with this, our study showed that D‐D and FIB levels decreased while PT increased, suggesting that the combined treatment exerted a regulatory effect on coagulation and fibrinolytic balance, which could contribute to improved placental perfusion and pregnancy stabilization [16].

This study provides several important and novel insights. First, it is one of the few clinical investigations to specifically evaluate JWSTW combined with dydrogesterone in women with TA complicated by ECF—a high‐risk subgroup seldom explored in previous literature. By targeting this particular population, the study addresses a clinically relevant gap concerning pregnancy maintenance in the presence of ECF. Second, the study’s design integrated TCM syndrome evaluation, endocrine and coagulation parameters, and ultrasound‐based ECF assessment, enabling a multidimensional evaluation of treatment efficacy. Third, the randomized allocation, blinded outcome assessment, and standardized follow‐up procedures enhanced the methodological rigor and reduced potential bias. Compared with earlier studies that primarily assessed STP or dydrogesterone alone, this study offers several advantages. The combined intervention demonstrated superior outcomes in symptom improvement, endocrine regulation, and effusion absorption without increasing adverse events, highlighting the potential synergistic effect of integrating herbal and hormonal therapy. Furthermore, by including biochemical and imaging indicators, the present study provides more objective evidence to support clinical efficacy than previous reports relying mainly on subjective symptom assessment.

Regarding generalizability, JWSTW is widely used and standardized in China but has not been approved by Western regulatory authorities such as the European Medicines Agency (EMA) or the U.K. Medicines and Healthcare Products Regulatory Agency (MHRA). Therefore, the findings are primarily applicable to countries or regions where TCM has been incorporated into clinical obstetric practice. Nonetheless, the underlying biological mechanisms observed—such as endocrine regulation, anti‐inflammatory modulation, and improvement of uterine microcirculation—may be relevant to global reproductive medicine and could inform future development of adjunctive therapies beyond TCM contexts.

The strengths of this study include a rigorous randomized design, blinded outcome evaluation, comprehensive outcome measures, and integration of both clinical and laboratory parameters. However, several limitations should be noted. This was a single‐center study with a modest sample size, potentially limiting external validity. In addition, the absence of a JWSTW‐only arm precludes a precise assessment of the herbal component’s independent contribution to efficacy. Finally, the relatively short follow‐up period prevented evaluation of long‐term pregnancy outcomes. Future research should aim to conduct multicenter, large‐sample randomized controlled trials to confirm these findings and evaluate long‐term maternal and fetal outcomes. Mechanistic studies focusing on molecular pathways—such as hormonal receptor regulation, inflammatory cytokine modulation, and vascular remodeling—would help elucidate the pharmacological basis of JWSTW. International collaborative studies conducted under ICH–GCP standards are also encouraged to assess safety and efficacy across diverse populations and to support potential global recognition of this TCM‐based therapy.

In summary, in Chinese clinical practice, JWSTW combined with dydrogesterone demonstrated favorable outcomes in women with TA complicated by ECF, showing benefits in symptom relief, endocrine regulation, and effusion absorption. The treatment appeared safe and well tolerated, offering a promising integrative approach for early pregnancy preservation. However, further rigorous, multiregional studies are needed to validate its generalizability and mechanistic basis.

## Funding

No funds, grants, or other support was received.

## Conflicts of Interest

The authors declare no conflicts of interest.

## Supporting Information

Supporting Table 1 Comparison of baseline characteristics between the two groups.

## Supporting information


**Supporting Information** Additional supporting information can be found online in the Supporting Information section.

## Data Availability

The data that support the findings of this study are available from the corresponding author upon reasonable request.
