# Supplementary material for: Efficacy of Jia Wei Shoutai Wan Combined With Dydrogesterone in the Treatment of Threatened Abortion Complicated With Endometrial Cavity Fluid: A Prospective, Single‐Center, Randomized Controlled Trial
Source: Int J Endocrinol. 2026 Apr 5;2026:5226490. doi: 10.1155/ije/5226490 (PMC13051817; doi:10.1155/ije/5226490)
Supplement: Supplementary file 1 — Supporting Information Additional supporting information can be found online in the Supporting Information section. [file IJE-2026-5226490-s001.docx]

**Supplementary Table 1** Comparison of baseline characteristics between the two groups

| Baseline characteristics | Control group (n = 50) | Combination group (n = 53) | *P* |
| --- | --- | --- | --- |
| Age (years) | 32.52 ± 5.22 | 31.96 ± 5.41 | 0.140 |
| Gestational week (week) | 8.04 ± 0.87 | 8.23 ± 0.79 | 0.248 |
| Gravidity (times) | 2 (1,3) | 2 (1,3) | 0.517 |
| Parity (times) | 0.5 (0,1) | 1 (0,1) | 0.337 |
| History of miscarriage [n(%)] | 3 (6.00) | 5 (9.43) | 0.515 |
| Duration of vaginal bleeding (d) | 3 (1,4) | 3 (1,5) | 0.461 |
